# Applications of Machine Learning to Diagnosis of Parkinson’s Disease

**DOI:** 10.3390/brainsci13111546

**Published:** 2023-11-03

**Authors:** Hong Lai, Xu-Ying Li, Fanxi Xu, Junge Zhu, Xian Li, Yang Song, Xianlin Wang, Zhanjun Wang, Chaodong Wang

**Affiliations:** 1 Department of Neurology, Xuanwu Hospital of Capital Medical University, National Clinical Research Center for Geriatric Diseases, Beijing 100053, China; laihong_0510@163.com (H.L.); lixuying_0325@163.com (X.-Y.L.); xufanxi@ccmu.edu.cn (F.X.); jungezhu9411@163.com (J.Z.); 18842663971@163.com (X.L.); song_yang_2004@sina.com (Y.S.); xianlingw6@sina.com (X.W.); wzjd421@163.com (Z.W.); 2 Department of Neurology, The First Affiliated Hospital of Gannan Medical University, Ganzhou 341000, China

**Keywords:** Parkinson’s disease, external validation, machine learning, support vector machine, diagnostic accuracy

## Abstract

Background: Accurate diagnosis of Parkinson’s disease (PD) is challenging due to its diverse manifestations. Machine learning (ML) algorithms can improve diagnostic precision, but their generalizability across medical centers in China is underexplored. Objective: To assess the accuracy of an ML algorithm for PD diagnosis, trained and tested on data from different medical centers in China. Methods: A total of 1656 participants were included, with 1028 from Beijing (training set) and 628 from Fuzhou (external validation set). Models were trained using the least absolute shrinkage and selection operator–logistic regression (LASSO-LR), decision tree (DT), random forest (RF), eXtreme gradient boosting (XGboost), support vector machine (SVM), and k-nearest neighbor (KNN) techniques. Hyperparameters were optimized using five-fold cross-validation and grid search techniques. Model performance was evaluated using the area under the curve (AUC) of the receiver operating characteristic (ROC) curve, accuracy, sensitivity (recall), specificity, precision, and F1 score. Variable importance was assessed for all models. Results: SVM demonstrated the best differentiation between healthy controls (HCs) and PD patients (AUC: 0.928, 95% CI: 0.908–0.947; accuracy: 0.844, 95% CI: 0.814–0.871; sensitivity: 0.826, 95% CI: 0.786–0.866; specificity: 0.861, 95% CI: 0.820–0.898; precision: 0.849, 95% CI: 0.807–0.891; F1 score: 0.837, 95% CI: 0.803–0.868) in the validation set. Constipation, olfactory decline, and daytime somnolence significantly influenced predictability. Conclusion: We identified multiple pivotal variables and SVM as a precise and clinician-friendly ML algorithm for prediction of PD in Chinese patients.

## 1. Introduction

Parkinson’s disease (PD) is the second most prevalent neurodegenerative disorder among the elderly population, primarily characterized by motor symptoms such as bradykinesia, rigidity, resting tremor, and postural instability. Alongside these, a range of non-motor symptoms (NMS) including constipation, hyposmia, REM sleep behavior disorders (RBD), depression, and cognitive impairment further complicate the clinical picture [1]. As the global population ages, PD prevalence continues to increase, affecting approximately 1.7% of individuals aged 65 years and older and 4.0% of those aged 80 years and older [2]. PD significantly impacts patients’ quality of life, social functioning, and family dynamics, imposing a substantial financial burden on individuals and society [3,4,5]. Current diagnostic criteria for PD primarily rely on clinical manifestations, and there is a lack of disease-modifying therapies available. This may be partly due to the loss of more than 50% of dopaminergic cells in the substantia nigra at the time of clinical diagnosis [6]. Early and accurate diagnosis of PD is crucial for implementing effective treatments and improving patient prognosis.

Extensive research has identified potential risk factors and protective factors for PD. Family history [7], pesticide exposure [8], occupational solvent exposure [8], and prodromal symptoms of PD [9] (including hyposmia, constipation, depression, rapid eye movement sleep behavior disorder (RBD), global cognitive deficit, daytime somnolence, and orthostatic hypotension) have been linked to an elevated risk of PD. Conversely, smoking [10], tea consumption [11], coffee intake [12], and physical activity [13] have been associated with a reduced risk of PD. Genome-wide association studies (GWAS) have identified numerous single-nucleotide polymorphisms (SNPs) at loci such as SNCA, GBA, LRRK2, PARK16, BST1, and MAPT which can modulate the risk of PD [14,15]. Leveraging these factors, high-risk populations can be identified, and targeted disease prevention measures can be deployed. However, an efficacious classification model that accurately discerns PD patients from healthy controls (HCs) remains elusive.

The advent of artificial intelligence has propelled machine learning (ML) to the forefront as an indispensable tool for disease diagnosis, progression evaluation, and prognosis assessment [16,17,18]. ML algorithms possess the potential to identify intricate data patterns, automate data analysis, and classify patient-specific data, which can be harnessed for precision medicine applications in PD. In recent years, ML has been applied to PD diagnosis using diverse data modalities, including speech and phonation evaluation [19], handwriting patterns [20], gait analysis [21], neuroimaging [22], cerebrospinal fluid (CSF) [23], and genetic and transcriptomic data [24].

Studies evaluating clinical and imaging biomarkers for the diagnosis of PD have been widely reported. For instance, Kang et al. [25] highlighted the prognostic and diagnostic potential of measures such as CSF Aβ1-42, T-tau, P-tau181, and α-synuclein in early-stage PD. Silveira-Moriyama et al. [26], through their research on smell identification tests in Brazil, underscored the importance of olfactory dysfunction in diagnosing PD. Additionally, Shinde et al. [27] employed neuromelanin-sensitive MRI to identify predictive markers for PD, while Armañanzas et al. [28] leveraged machine learning to pinpoint significant non-motor symptoms associated with PD severity. However, many studies have focused solely on individual biomarkers or imaging indicators, neglecting the potential of integrating diverse data types. Furthermore, the application of advanced machine learning techniques in PD research, despite their proven efficacy in other domains, remains underexplored. These gaps underscore the need for a more comprehensive approach, which our study aims to address by enhancing PD diagnostic accuracy through diverse machine learning algorithms, particularly tailored for the Chinese population.

The crucial questions for ML-based diagnostic models for PD include: what the pivotal variables are for PD, what the best ML algorithm is in the model construction, and how the models perform in different cohorts. To address these questions, we performed a comprehensive analysis using multi-faceted variables including demographic data, environmental factors, lifestyle habits, NMS, and genetic factors, constructed the models using six algorithms, and validated the models using a dataset from a separate (southern) population. Our research pinpointed several key variables and identified SVM as the most accurate and user-friendly ML algorithm for the prediction of PD in the Chinese population. We then present a more precise population. Consequently, we introduce a refined and pragmatical diagnostic model, providing a novel perspective for both the research and treatment of Parkinson’s.

## 2. Methods

### 2.1. Study Design and Data Source

**Figure 1:**
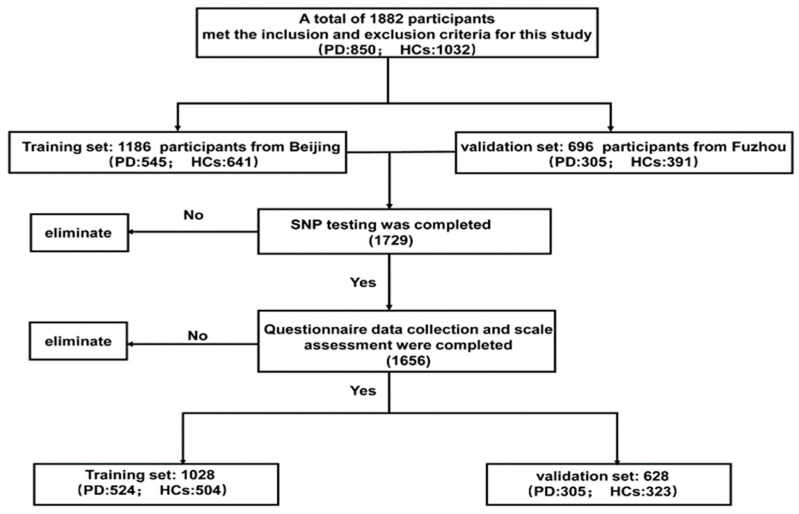
Flow diagram of selection of patients.

The study was designed for training and validation tests. The study was approved by the Medical Ethics Committee of Xuanwu Hospital of Capital Medical University. Informed consent was obtained from all participants in the study. Two populations of participants were included: a training set to train the algorithms and a validation set to independently evaluate each algorithm’s performance. PD patients in the training set were recruited from Xuanwu Hospital of Capital Medical University, while those in the validation set were recruited from Fujian Medical University Union Hospital. Concurrently, healthy controls (HCs) in the training set were selected from among the residents of Beijing, and HCs in the validation set were recruited from Fuzhou communities, matching proportionally (within 5-year strata) to cases by gender and age. Patients were diagnosed by senior neurologists specializing in movement disorders based on the MDS clinical diagnostic criteria for PD released in 2015 [29]. Patients were excluded from the study using the following criteria: (i) Parkinson’s plus syndromes, including multiple system atrophy (MSA), progressive supranuclear palsy (PSP), dementia with Lewy bodies (DLB), and corticobasal degeneration (CBD); (ii) Parkinsonian syndromes caused by cerebrovascular diseases, brain trauma, hypoxic diseases, infectious diseases, and metabolic diseases, affecting the central nervous system; (iii) malignant tumors or other serious systemic diseases; and (iv) dementia, rendering them unable to cooperate with the questionnaire. The control participants who were enrolled fulfilled the following criteria: (i) lack of PD-related motor symptoms (bradykinesia, tremor, postural instability and rigidity); (ii) no history of dementia, PD, or other neurodegenerative diseases; and (iii) no history of malignant tumors or other serious systemic diseases. A total of 1882 participants were recruited from December 2016 to September 2021. After excluding 226 participants due to incomplete gene detection or critical assessments, we had a final count of 1656 participants. The training set comprised 1028 individuals (524 PD patients and 504 HCs), while the external validation set included 628 individuals (305 PD patients and 323 HCs) (Figure 1). All participants were assessed for demographic information, lifestyle behaviors, and environmental exposures. Participants were also assessed for motor and NMS of PD using diagnostic scales (additional information provided in Supplementary Methods). All assessments were performed via face-to-face interviews by clinical investigators with unified training.

### 2.2. Candidate Variables

The accuracy of any model largely depends on the variables it considers. Here, we outline the variables chosen in our study. Drawing on the previously published literature [2,30,31], we selected 26 potential predictive variables for inclusion in the prediction model: demographic variables (age, sex, and education level), family history of PD, head injury with unconsciousness, general anesthesia, PD-related NMS (possible RBD, olfactory dysfunction, constipation, global cognitive deficit, depression, and daytime somnolence), lifestyle factors (smoking, alcohol consumption, and tea and coffee intake), environmental exposures (pesticides, organic solvents, and heavy metals), as well as 7 PD-associated SNPs in Chinese and other populations (MMRN1 rs6532194, RAB7L1 rs823144, SNCA rs356182 and rs356219, LRRK2 rs34778348, MCCC1 rs12637471, and GBA rs421016) [32,33,34,35,36,37].

### 2.3. Genotype Analysis and Classification

Genetic factors play a pivotal role in many diseases, including Parkinson’s. This subsection elaborates on the genotype analysis we conducted. Genomic DNA was extracted from peripheral blood leukocytes using a standard protocol. Polymerase chain reaction (PCR) assays and extension primers for variants were designed employing the MassARRAY Assay Design software version 4.0 (Sequenom, Inc., San Diego, CA, USA) and the Primer 6.0 software (Premier, Vancouver, BC, Canada). The primer sequences for amplifying each SNP are listed in Supplementary Table S1. PCR products were purified and sequenced using an ABI3730xl DNA analyzer (Applied Biosystems, Inc., Waltham, MA, USA). Sequence readings were performed with the Chromas 2.22 software.

### 2.4. Machine Learning Algorithm

Machine learning stands as the cornerstone of our research. In this section, we delve into the algorithms we have harnessed and the metrics we utilize for evaluation. Six supervised ML algorithms were employed: least absolute shrinkage and selection operator–logistic regression (LASSO-LR), decision tree (DT), random forest (RF), eXtreme gradient boosting (XGboost), support vector machine (SVM), and k-nearest neighbor (KNN). In the training set, we applied five-fold cross-validation to minimize each model’s overfitting and utilized the grid search technique to select the optimal combination of hyperparameters for each ML algorithm. Algorithm performances were evaluated based on the area under the curve (AUC) of the receiver operating characteristic (ROC) curve, accuracy, sensitivity (recall), specificity, precision, and F1 score. The model with the highest accuracy was chosen for evaluation using the validation set. In addition to performance comparisons, we also analyzed the importance of variable factors in the models to identify which variables were critical in distinguishing PD patients from HCs in the training set. While deep learning techniques have shown significant promise in various applications, we opted not to use them in this study due to several considerations. Firstly, deep learning models typically require large datasets for effective training and to mitigate overfitting. Secondly, the intricacies of parameter tuning and model selection can be challenging. Furthermore, the complexity of deep learning models might compromise the interpretability of results, which was a key focus for our study. Given our dataset’s size and our emphasis on clear variable interpretation, we chose the machine learning algorithms described above. Detailed information about the ML algorithms used in this study is provided in the Supplementary Materials.

### 2.5. Statistical Analysis

This subsection dives into the statistical techniques we used to validate and interpret our findings. All statistical analyses were performed using the R software (version 4.1.1; R Foundation for Statistical Computing Vienna, Austria; http://www.R-project.org/, accessed on 9 October 2023). Categorical variables were expressed as frequencies and percentages, while continuous variables were described as medians and interquartile ranges (IQRs). Mann–Whitney U tests (for continuous variables with skewed distributions) and the chi-square test (for categorical variables) were utilized to compare differences in characteristics between groups. The R package “glmnet” was employed to perform the LASSO regression. The R packages “pROC”, “plotROC”, and “rmda” were applied to generate the ROC. The R package “caret” was utilized to run the ML algorithms. All tests were two-tailed, and *p* < 0.05 was defined as statistically significant.

## 3. Results

### 3.1. Clinical and Demographic Characteristics

Here, we present an overview of the clinical and demographic traits of our dataset. In the training set, the median age for PD patients was 66 years (interquartile range, 61–71.25 years), with 49% being male. The HCs had a median age of 67 years (interquartile range, 63–70 years), with 45% male representation. In the external validation set, PD patients had a median age of 69 years (interquartile range, 61–75 years), with 56.7% being male, while HCs had a median age of 68 years (interquartile range, 65–73 years), with 50.2% being male. Detailed demographic and clinical characteristics of the training and validation sets are provided in Table 1.

### 3.2. Comparison of Algorithms

**Figure 2:**
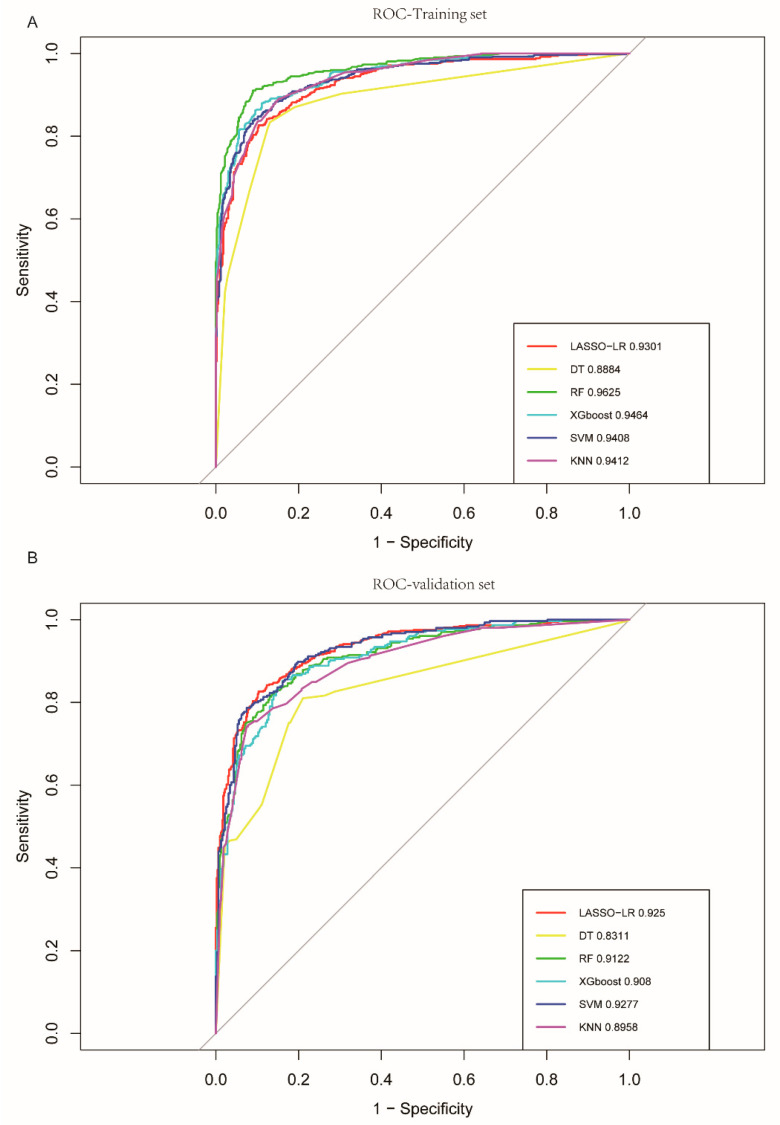
Receiver operating characteristic (ROC) curve of LASSO-LR, decision tree, random forest, XGboost, SVM, and KNN for the training set and validation set. (**A**) Integration of ROC for the PD classification model based on the six models for the training set. (**B**) Integration of ROC for the PD classification model based on the six models for the validation set.

With multiple algorithms being considered, it is essential to understand their comparative strengths and weaknesses. This section provides a side-by-side analysis of the algorithms, highlighting their performance metrics. Table 2 presents the performances of various ML algorithms with the training and validation sets, while Figure 2A,B illustrates the ROC of all six models with the training set and validation set.

LASSO–logistic regression: Utilizing 10-fold cross-validation, a LASSO regression model was employed to select predictive variables from among the preliminary factors. Following LASSO regression selection (Supplementary Figure S1), 15 variables with non-zero coefficients that minimized the overall lambda were identified as potential optimal variables for distinguishing PD. The optimal value of lambda, the ridge penalty, was 0.021. The tuned model in the training set accurately classified 433 out of 524 PD patients and 452 out of 504 HCs, achieving an AUC of 0.930 (95% CI: 0.915–0.945), an F1 score of 0.858 (95% CI: 0.834–0.879), and an overall accuracy of 0.861 (95% CI: 0.840–0.881). In the validation set, the model accurately classified 247 out of 305 PD patients and 282 out of 323 HCs, yielding an AUC of 0.925 (95% CI: 0.905–0.945), an F1 score of 0.833 (95% CI: 0.799–0.864), and an overall accuracy of 0.842 (95% CI: 0.812–0.869).

Decision tree: Employing five-fold cross-validation and the grid search method (Supplementary Figure S2A), the optimal complexity parameter (CP) for DT was determined to be 0.006. The tuned model in the training set accurately classified 437 out of 524 PD patients and 438 out of 504 HCs, resulting in an AUC of 0.888 (95% CI: 0.868−0.909), an F1 score of 0.851 (95% CI: 0.828–0.875), and an overall accuracy of 0.851 (95% CI: 0.829–0.873) In the validation set, the model accurately classified 229 out of 305 PD patients and 265 out of 323 HCs, yielding an AUC of 0.831 (95% CI: 0.80–0.862), an F1 score of 0.774 (95% CI: 0.731–0.811), and an overall accuracy of 0.787 (95% CI: 0.753–0.820).

Random forest: Utilizing five-fold cross-validation and the grid search method (Supplementary Figure S2B), 500 decision trees and three predictor variables selected at each node (mtry) provided optimal performance. In the training set, the tuned model accurately classified 477 out of 524 PD patients and 459 out of 504 HCs, achieving an AUC of 0.963 (95% CI: 0.952–0.973), an F1 score of 0.912 (95% CI: 0.894–0.930), and an overall accuracy of 0.911(95% CI: 0.892–0.928). In the validation set, the tuned model accurately classified 264 out of 305 PD patients and 260 out of 323 HCs, yielding an AUC of 0.912 (95% CI: 0.890–0.934), an F1 score of 0.835 (95% CI: 0.802–0.867), and an overall accuracy of 0.834 (95% CI: 0.804–0.863).

EXtreme gradient boosting: Employing five-fold cross-validation and the grid search method, the optimal parameters for the XGBoost model were as follows: learning rate (eta) of 0.3, colsample_bytree of 0.6, gamma of 0, maximum tree depth (max_depth) of 1, subsample of 0.875, and minimum leaf node sample weight (min_child_weight) of 1 (Supplementary Figure S2C). In the training set, the tuned model accurately classified 461 out of 524 PD patients and 448 out of 504 HCs, achieving an AUC of 0.946 (95% CI: 0.946–0.959), an F1 score of 0.886 (95% CI: 0.866–0.905), and an overall accuracy of 0.884 (95% CI: 0.865–0.904). In the validation set, the tuned model accurately classified 271 out of 305 PD patients and 243 out of 323 HCs, yielding an AUC of 0.908 (95% CI: 0.886–0.931), an F1 score of 0.826 (95% CI: 0.794–0.858), and an overall accuracy of 0.819 (95% CI: 0.788–0.849).

Support vector machine: A five-fold cross-validation and grid search determined that the optimal SVM cost choice C was 0.25, and the optimal kernel smoothing parameter σ was 0.04 (Supplementary Figure S2D). In the training set, the tuned model accurately classified 439 out of 524 PD patients and 459 out of 504 HCs, achieving an AUC of 0.941 (95% CI: 0.927–0.955), an F1 score of 0.871 (95% CI: 0.849–0.895), and an overall accuracy of 0.874 (95% CI: 0.854–0.896). In the validation set, the tuned model accurately classified 252 out of 305 PD patients and 278 out of 323 HCs, yielding an AUC of 0.928 (95% CI: 0.908–0.947), an F1 score of 0.837 (95% CI: 0.803–0.868), and an overall accuracy of 0.844 (95% CI: 0.814–0.871).

K-nearest neighbor: Based on five-fold cross-validation and grid search (Supplementary Figure S2E), the optimal number of nearest neighbors (k) was found to be nine. In the training set, the tuned model accurately classified 467 out of 524 PD patients and 427 out of 504 HCs, achieving an AUC of 0.941 (95% CI: 0.928–0.954), an F1 score of 0.875 (95% CI: 0.854–0.896), and an overall accuracy of 0.870 (95% CI: 0.849–0.890). In the validation set, the tuned model accurately classified 253 out of 305 PD patients and 256 out of 323 HCs, yielding an AUC of 0.896 (95% CI: 0.871–0.921), an F1 score of 0.810 (95% CI:0.774–0.842), and an overall accuracy of 0.811 (95% CI: 0.779–0.841).

**Figure 3:**
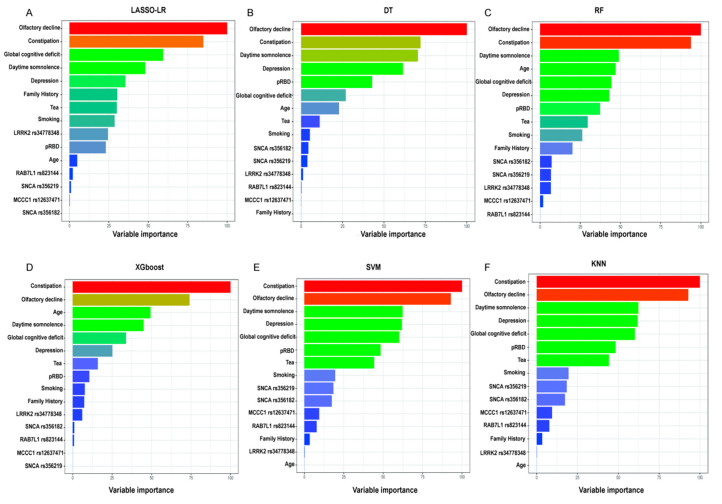
Variable importance. Histograms (**A**–**F**) depict the proportion of factor importance for different predictors in the model. For each model, the relative importance is quantified by assigning a weight between 0 and 1 for each variable.

Variable importance: The significance of features, as demonstrated by effect sizes, was calculated (Figure 3A–F). Olfactory dysfunction and constipation exhibited the highest frequencies among the top predictors across all six models, while daytime somnolence, global cognitive deficit, and depression also displayed large effect sizes in more than half of the models.

The SVM model, exhibiting the most remarkable discrimination capabilities, outperformed other models by achieving the highest AUC, overall accuracy, and F1 score in the validation set.

## 4. Discussion

The primary objective of this study was to develop a non-invasive, cost-effective, and accurate classification model to differentiate patients with PD and HCs by employing genetic factors, lifestyle factors, environmental exposures, and NMS. The current study combined analyses of multiple variable types and ML algorithms, and the model validation used datasets from distinct geographic centers. The results help to establish a comprehensive approach to the early and precise diagnosis of PD for the Chinese population.

Previous studies have typically relied on individual features for PD prediction [25,26,27,28]. However, models incorporating multiple factors have demonstrated increased accuracy compared to those based on single risk factors. In recent years, the application of ML algorithms has gained increasing attention. Table 3 [38,39,40,41,42] provides a comparison of several models. R. Prashanth et al. utilized a combination of NMS features, cerebrospinal fluid (CSF), and imaging markers to differentiate early PD subjects from normal individuals using the naïve Bayes, SVM, boosted trees, and RF methods, observing that the SVM classifier delivered the most optimal performance [38]. Govindu et al. employed machine learning techniques in telemedicine, using classifiers such as SVM, RF, KNN, and logistic regression on collected audio data. Among these, the random forest (RF) classifier stood out, achieving a remarkable 91.83% accuracy in early PD detection [42]. However, many existing models generated overfitting, especially when they were trained on limited datasets. Additionally, the unclear interpretability of some complex models makes it challenging for clinicians to trust and adopt these models in practice. Furthermore, several models were constructed using isolated datasets, and their generalizability across diverse populations and medical centers has yet to be thoroughly evaluated.

Our study addressed some of these gaps by evaluating and comparing multiple ML algorithms for PD detection, emphasizing both performance and interpretability. First, our data originated from different medical centers, which not only adds diversity to the datasets but also enhances the model’s generalizability. Second, in contrast to studies that rely on expensive radiomics or invasive cerebrospinal fluid collection, our approach is non-invasive and cost-effective. This holds tremendous potential for large-scale screening and clinical applications. Moreover, our model took into account a wider range of factors, including genetic factors, lifestyle factors, environmental exposure, and non-motor symptoms, contributing to a more accurate diagnosis of PD. Lastly, our data collection process was straightforward and user-friendly, and thus is easier for clinical doctors. In summary, our study offered a cost-effective, non-invasive, comprehensive, and easily accessible approach to the prediction of PD, and it is suitable for large-scale screening and clinical practice.

Currently, researchers are exploring novel biomarkers for diagnosing and predicting PD [43,44,45,46]. Although numerous studies have been conducted, no widely recognized and easy-to-use predictive tool currently exists. In this study, we employed ML algorithms and discovered that genetic factors, lifestyles, environmental exposures, and NMS serve as robust predictors of PD, largely aligning with previous findings [2,47]. Notably, we identified a strong association between PD and NMS. The variable importance analysis of the six ML models demonstrated that NMS were among the top predictors with the highest frequencies.

At present, the diagnosis of PD predominantly relies on clinical motor symptoms; however, NMS could potentially serve as an early warning for PD progression, as they may precede motor symptoms by decades [48]. This period, known as the prodromal stage, is crucial for identifying individuals with early-stage PD, potentially paving the way for disease-modifying treatments that could delay or even prevent the progression to manifest Parkinson’s disease if administered early.

Among the prodromal PD indicators in our study, olfactory dysfunction emerged as the strongest association, followed by constipation and daytime somnolence. This relationship aligns with previous findings on PD risk. Olfactory dysfunction, one of the most common and typical NMS associated with PD [49], is found in approximately 50–90% of PD patients [50], often manifesting as one of the disease’s earliest symptoms [51]. Most patients develop olfactory dysfunction 4–6 years before the onset of motor impairment [47]. This is supported by Braak et al., who identified the presence of Lewy bodies and Lewy neurites in the olfactory bulb during the premotor stage of PD [52]. Constipation, a marker of early PD, affects approximately half of patients before the emergence of overt motor symptoms [53]. The neuropathology of α-synuclein in the enteric nervous system may precede typical changes in the midbrain and limbic regions [49]. Prior research has shown a higher prevalence of daytime somnolence in PD (35.1%) compared to the general population (9.0–16.8%) [54]. The Honolulu–Asia Aging study revealed that men reporting subjective daytime somnolence had a 2.8-fold increased relative risk of developing PD [55]. Patients newly diagnosed with PD may already exhibit mild cognitive deficits, with nearly 50% developing PD dementia within 10 years of disease onset [56]. Depression, one of the earliest NMS in PD, has an incidence in PD patients three times higher than in the general population [57]. A large-scale retrospective cohort analysis of 32,415 individuals suggested that most patients developed depression approximately 10 years before motor symptoms [58]. RBD is a frequent and significant prodromal symptom of PD [59]. Approximately 50% of idiopathic RBD patients convert to PD within a decade, and ultimately, over 80% develop some form of neurodegenerative disease [59,60].

NMS bear clinical significance for early diagnosis. For instance, PD patients in the prodromal phase may present with NMS such as sleep disorders, olfactory dysfunction, mood disorders, constipation, and limb and trunk pain, but lack overt motor symptoms. These patients often visit sleep clinics, otorhinolaryngology clinics, psychological clinics, gastroenterology clinics, and orthopedics clinics, leading to delayed diagnosis. Furthermore, patients with limb pain may be mistakenly diagnosed with cervical or lumbar issues, and surgical intervention often fails to alleviate the pain. Employing an ML model can aid non-neurological clinicians in rapidly distinguishing PD patients from HCs, facilitating timely referrals to neurology departments for professional diagnosis and treatment.

There are several limitations to this study. First, as with all retrospective studies, potential selection biases and unknown confounding factors may not be accounted for in the analysis. To address these limitations, future work will include a prospective cohort study with a larger sample size. Second, as only one center was used for external validation, a larger sample size from multiple research centers is necessary to verify and improve the study’s results. Third, due to time and resource constraints, we were unable to examine the participants’ imaging indicators.

## 5. Conclusions

In conclusion, we identified multiple pivotal variables and SVM as a precise and clinician-friendly ML algorithm for the prediction of PD in Chinese patients. We believe that these ML algorithms, with further optimization, can serve as invaluable adjuncts to clinicians in the PD diagnostic process, potentially facilitating earlier and more accurate detection of the disease.

## Figures and Tables

**Table 1:** Characteristics of total participants.

	Training Set (*n* = 1028)		*p*	Validation Set (*n* = 628)		*p*
	PD	HC		PD	HC	
	(*n* = 524)	(*n* = 504)		(*n* = 305)	(*n* = 323)	
Demographics						
Age, year	66 (61, 71.25)	67 (63, 70)	0.222	69 (61, 75)	68 (65, 73)	0.159
Male	257 (49)	227 (45)	0.221	173 (56.7)	162 (50.2)	0.117
Education, year	9 (5, 11.12)	9 (9, 12)	<0.001	8 (5, 11)	9 (6, 11)	0.001
Family History of PD	44 (8.4)	12 (2.4)	<0.001	27 (8.9)	3 (0.9)	<0.001
Lifestyle behaviors						
Smoking	124 (23.7)	188 (37.3)	<0.001	82 (26.9)	121 (37.5)	0.006
Alcohol	175 (33.4)	209 (41.5)	0.009	62 (20.3)	145 (44.9)	<0.001
Tea	203 (38.7)	323 (64.1)	<0.001	57 (18.7)	213 (65.9)	<0.001
Coffee	66 (12.6)	93 (18.5)	0.012	8 (2.6)	32 (9.9)	<0.001
Environmental exposure						
Pesticides	157 (30)	110 (21.8)	0.004	91 (29.8)	80 (24.8)	0.181
Organic solvent	13 (2.5)	18 (3.6)	0.401	5 (1.6)	10 (3.1)	0.351
Heavy metal	12 (2.3)	18 (3.6)	0.301	4 (1.3)	6 (1.9)	0.753
Head injury	21 (4)	12 (2.4)	0.193	2 (0.7)	6 (1.9)	0.288
General anesthesia	105 (20)	117 (23.2)	0.245	44 (14.4)	51 (15.8)	0.715
Non-motor symptoms						
pRBD	172 (32.8)	28 (5.6)	<0.001	75 (24.6)	12 (3.7)	<0.001
Olfactory dysfunction	319 (60.9)	63 (12.5)	<0.001	217 (71.1)	38 (11.8)	<0.001
Constipation	347 (66.2)	73 (14.5)	<0.001	169 (55.4)	54 (16.7)	<0.001
Global cognitive deficit	273 (52.1)	97 (19.2)	<0.001	185 (60.7)	103 (31.9)	<0.001
Depression	242 (46.2)	63 (12.5)	<0.001	44 (14.4)	17 (5.3)	<0.001
Daytime somnolence	216 (41.2)	37 (7.3)	<0.001	161 (52.8)	47 (14.6)	<0.001
Gene mutation						
MMRN1 rs6532194	208 (39.7)	143 (28.4)	<0.001	106 (34.8)	105 (32.5)	0.609
RAB7L1 rs823144	192 (36.6)	144 (28.6)	0.007	127 (41.6)	94 (29.1)	0.001
SNCA rs356182	405 (77.3)	326 (64.7)	<0.001	248 (81.3)	182 (56.3)	<0.001
LRRK2 rs34778348	65 (12.4)	40 (7.9)	0.024	23 (7.5)	23 (7.1)	0.961
SNCA rs356219	208 (39.7)	134 (26.6)	<0.001	109 (35.7)	98 (30.3)	0.176
MCCC1 rs12637471	352 (67.2)	294 (58.3)	0.004	218 (71.5)	182 (56.3)	<0.001
GBA rs421016	7 (1.3)	0 (0)	0.015	5 (1.6)	0 (0)	0.027

Abbreviations: pRBD = possible REM sleep behavior disorder; HC = healthy control. Values are *n* (%) or medians and interquartile range (IQRs).

**Table 2:** A comparison of the model performances with the training and validation set.

	**LASSO-LR**	**DT**	**RF**	**XGboost**	**SVM**	**KNN**
Training set						
AUC	0.930 (95% CI: 0.915–0.945)	0.888 (95% CI: 0.868–0.909)	0.963 (95% CI: 0.952 −0.973)	0.946 (95% CI: 0.946–0.959)	0.941 (95% CI: 0.927–0.955)	0.941 (95% CI: 0.928–0.954)
Accuracy	0.861 (95% CI: 0.840–0.881)	0.851 (95% CI: 0.829–0.873)	0.911 (95% CI: 0.892–0.928)	0.884 (95% CI: 0.865–0.904)	0.874 (95% CI: 0.854–0.896)	0.870 (95% CI: 0.849–0.890)
Sensitivity (Recall)	0.826 (95% CI: 0.789–0.860)	0.834 (95% CI: 0.803–0.866)	0.910 (95% CI: 0.884–0.936)	0.880 (95% CI: 0.850–0.909)	0.838 (95% CI: 0.805–0.872)	0.891 (95% CI: 0.863–0.919)
Specificity	0.897 (95% CI: 0.870–0.924)	0.869 (95% CI: 0.840–0.898)	0.911 (95% CI: 0.884–0.936)	0.889 (95% CI: 0.860–0.916)	0.911 (95% CI: 0.885–0.936)	0.847 (95% CI: 0.817–0.880)
Precision	0.893 (95% CI: 0.866–0.919)	0.869 (95% CI: 0.839–0.897)	0.914 (95% CI: 0.890–0.937)	0.892 (95% CI: 0.867–0.918)	0.907 (95% CI: 0.882–0.933)	0.859 (95% CI: 0.831–0.888)
F1 score	0.858 (95% CI: 0.834–0.879)	0.851 (95% CI: 0.828–0.875)	0.912 (95% CI: 0.894–0.930)	0.886 (95% CI: 0.866–0.905)	0.871 (95% CI: 0.849–0.895)	0.875 (95% CI: 0.854–0.896)
Validation set						
AUC	0.925 (95% CI: 0.905–0.945)	0.831 (95% CI: 0.80–0.862)	0.912 (95% CI: 0.890–0.934)	0.908 (95% CI: 0.886–0.931)	0.928 (95% CI: 0.908–0.947)	0.896 (95% CI: 0.871–0.921)
Accuracy	0.842 (95% CI: 0.812–0.869)	0.787 (95% CI: 0.753–0.820)	0.834 (95% CI: 0.804–0.863)	0.819 (95% CI: 0.788–0.849)	0.844 (95% CI: 0.814–0.871)	0.811 (95% CI: 0.779–0.841)
Sensitivity	0.810 (95% CI: 0.764–0.853)	0.751 (95% CI: 0.701–0.80)	0.866 (95% CI: 0.826–0.902)	0.889 (95% CI: 0.851–0.922)	0.826 (95% CI: 0.786–0.866)	0.830 (95% CI: 0.779–0.841)
Specificity	0.873 (95% CI: 0.834–0.909)	0.820 (95% CI: 0.777–0.862)	0.805 (95% CI: 0.764–0.848)	0.752 (95% CI: 0.707–0.799)	0.861 (95% CI: 0.820–0.898)	0.793 (95% CI: 0.747–0.835)
Precision	0.858 (95% CI: 0.816–0.90)	0.798 (95% CI: 0.751–0.845)	0.807 (95% CI: 0.765–0.851)	0.772 (95% CI: 0.727–0.819)	0.849 (95% CI: 0.807–0.891)	0.791 (95% CI: 0.746–0.838)
F1 score	0.833 (95% CI: 0.799–0.864)	0.774 (95% CI: 0.731–0.811)	0.835 (95% CI: 0.802–0.867)	0.826 (95% CI: 0.794–0.858)	0.837 (95% CI: 0.803–0.868)	0.810 (95% CI: 0.774–0.842)

Abbreviations: LASSO-LR = least absolute shrinkage and selection operator–logistic regression; DT = decision tree; RF = random forest; XGboost = eXtreme gradient boosting; SVM = support vector machine; KNN = k-nearest neighbor; AUC = area under the curve of the receiver operating characteristic curve; CI = confidence interval.

**Table 3:** Comparative Analysis of Parkinson’s Disease Diagnosis Using Machine Learning Approaches.

Reference	Modeling Approach	Features Selection	Objective	Source of Data	No. of Subjects	Evaluation
R. Prashanth et al. [38]	Naïve Bayes, SVM, Boosted Trees, RF	NMS, CSF, dopaminergic imaging markers	Classification of PD from HC	PPMI	401 PD + 183 HC	Best performing: SVM with Accuracy = 96.40%, Sensitivity = 97.03%, Specificity = 95.01%, AUC = 98.88%.
Shu et al. [39]	Nomogram	MRI, clinical characteristics, NMS	Identification of early-stage PD	PPMI	168 PD + 168HC + atypical PD 58	AUC of training, testing and verification sets were 0.937, 0.922, and 0.836, respectively; the specificity were 83.8, 88.2, and 91.38%, respectively; and the sensitivity were 84.6, 82.4, and 70.69%.
Karabayir et al. [40]	Light Gradient Boosting	Questionnaires; simple non-invasive clinical tests	To predict a future diagnosis of PD	HAAS	292 subjects	Individuals who developed PD within 3 years: AUC = 0.82, (95% CI 0.76–0.89), 5 years: AUC = 0.77 (95% CI 0.71–0.84).
Lin et al. [41]	SVM RF	Gathering gait and postural transition data using wearable sensors.	To diferentiate early-stage PD from ET	Ruijin Hospital, Shanghai Jiao Tong University School of Medicine	84 early-stage PD and 80 ET subjects	Best performing: weighted average ensemble classifcation models with accuracy = 84%, sensitivity = 85.9%, Specifcity = 82.1%, AUC = 0.912.
Govindu et al. [42]	SVM, RF, KNN, Logistic Regression models	MDVP audio data	Early detection of PD	PPMI	23PD + 8 HC	Best performing: RF with accuracy = 91.83%, sensitivity = 0.95.

Abbreviations: PD = Parkinson’s disease; HC = healthy control; CSF = Cerebrospinal fluid; NMS = nonmotor symptoms; SVM = support vector machine; RF = random forest; AUC = area under the curve; PPMI = Parkinson’s Progress Markers Initiative; HAAS = Honolulu–Asia Aging Study; ET = essential tremor; KNN = k-nearest neighbors.

## Data Availability

The raw data supporting the conclusions of this article will be made available by the authors, without undue reservation.

## Supplementary Materials

The following supporting information can be downloaded at: https://www.mdpi.com/article/10.3390/brainsci13111546/s1. Table S1: The primer sequences for amplifying each SNPs. Figure S1: Feature selection using Lasso regression. (A) Lasso coefficient profiles of the candidate features. (B) Selection of the optimal parameter (lambda) through 10-fold cross-validation. The left and right dotted vertical lines represent the optimal lambda values using the minimum error criterion and one standard error (1-SE) of the minimum criterion, respectively. Figure S2: Hyperparameter optimization for each machine learning model using five-fold cross-validation and a grid search technique. (A) Hyperparameter optimization for the decision tree model. (B) Hyperparameter optimization for the random forest model. (C) Hyperparameter optimization for the eXtreme gradient boosting model. (D) Hyperparameter optimization for the support vector machine model. (E) Hyperparameter optimization for the k-nearest neighbor model.

## Abbreviations

PD: Parkinson’s disease; REM: rapid eye movement; RBD: REM sleep behavior disorder; NMS: non-motor symptoms; SNPs: single-nucleotide polymorphisms; iRBD: idiopathic RBD; pRBD: possible REM sleep behavior disorder; GWAS: genome-wide association studies; CI: confidence interval; OR: odds ratio; IQRs: interquartile ranges; RSS: residual sum of squares; Lasso: least absolute shrinkage and selection operator; ROC: receiver operating characteristic; AUC: area under the ROC curve; DCA: decision curve analysis; NRI: net reclassification improvement; IDI: integrated discrimination improvement; ML: machine learning; DT: decision tree; RF: random forest; XGboost: eXtreme gradient boosting; SVM: support vector machine; KNN: k-nearest neighbor; HCs: healthy controls; MSA: multiple system atrophy; PSP: progressive supranuclear palsy; DLB: dementia with Lewy bodies; CBD: corticobasal degeneration; PCR: polymerase chain reaction.

## References

[1] De Lau L.M.L., Breteler M.M.B. (2006). Epidemiology of Parkinson’s disease. Lancet Neurol..

[2] Ascherio A., Schwarzschild M.A. (2016). The epidemiology of Parkinson’s disease: Risk factors and prevention. Lancet Neurol..

[3] Yang J.-X., Chen L. (2017). Economic Burden Analysis of Parkinson’s Disease Patients in China. Park. Dis..

[4] Johnson S.J., Diener M.D., Kaltenboeck A., Birnbaum H.G., Siderowf A.D. (2013). An economic model of Parkinson’s disease: Implications for slowing progression in the United States. Mov. Disord. Off. J. Mov. Disord. Soc..

[5] Wilczyński J., Ścipniak M., Ścipniak K., Margiel K., Wilczyński I., Zieliński R., Sobolewski P. (2021). Assessment of Risk Factors for Falls among Patients with Parkinson’s Disease. BioMed Res. Int..

[6] Fearnley J.M., Lees A.J. (1991). Ageing and Parkinson’s disease: Substantia nigra regional selectivity. Brain J. Neurol..

[7] Rocca W.A., McDonnell S.K., Strain K.J., Bower J.H., Ahlskog J.E., Elbaz A., Schaid D.J., Maraganore D.M. (2004). Familial aggregation of Parkinson’s disease: The Mayo Clinic family study. Ann. Neurol..

[8] Pezzoli G., Cereda E. (2013). Exposure to pesticides or solvents and risk of Parkinson disease. Neurology.

[9] Heinzel S., Berg D., Gasser T., Chen H., Yao C., Postuma R.B., The MDS Task Force on the Definition of Parkinson’s Disease (2019). Update of the MDS research criteria for prodromal Parkinson’s disease. Mov. Disord. Off. J. Mov. Disord. Soc..

[10] Noyce A.J., Bestwick J.P., Silveira-Moriyama L., Hawkes C.H., Giovannoni G., Lees A.J., Schrag A. (2012). Meta-analysis of early nonmotor features and risk factors for Parkinson disease. Ann. Neurol..

[11] Hu G., Bidel S., Jousilahti P., Antikainen R., Tuomilehto J. (2007). Coffee and tea consumption and the risk of Parkinson’s disease. Mov. Disord. Off. J. Mov. Disord. Soc..

[12] Liu R., Guo X., Park Y., Huang X., Sinha R., Freedman N.D., Hollenbeck A.R., Blair A., Chen H. (2012). Caffeine Intake, Smoking, and Risk of Parkinson Disease in Men and Women. Am. J. Epidemiol..

[13] Fang X., Han D., Cheng Q., Zhang P., Zhao C., Min J., Wang F. (2018). Association of Levels of Physical Activity With Risk of Parkinson Disease: A Systematic Review and Meta-analysis. JAMA Netw. Open.

[14] Foo J.N., Chew E.G.Y., Chung S.J., Peng R., Blauwendraat C., Nalls M.A., Mok K.Y., Satake W., Toda T., Chao Y. (2020). Identification of Risk Loci for Parkinson Disease in Asians and Comparison of Risk between Asians and Europeans: A Genome-Wide Association Study. JAMA Neurol..

[15] Nalls M.A., Blauwendraat C., Vallerga C.L., Heilbron K., Bandres-Ciga S., Chang D., Tan M., Kia D.A., Noyce A.J., Xue A. (2019). Identification of novel risk loci, causal insights, and heritable risk for Parkinson’s disease: A meta-analysis of genome-wide association studies. Lancet Neurol..

[16] Kawakami E., Tabata J., Yanaihara N., Ishikawa T., Koseki K., Iida Y., Saito M., Komazaki H., Shapiro J.S., Goto C. (2019). Application of Artificial Intelligence for Preoperative Diagnostic and Prognostic Prediction in Epithelial Ovarian Cancer Based on Blood Biomarkers. Clin. Cancer Res. Off. J. Am. Assoc. Cancer Res..

[17] Angraal S., Mortazavi B.J., Gupta A., Khera R., Ahmad T., Desai N.R., Jacoby D.L., Masoudi F.A., Spertus J.A., Krumholz H.M. (2020). Machine Learning Prediction of Mortality and Hospitalization in Heart Failure with Preserved Ejection Fraction. JACC Heart Fail..

[18] Wang H.-H., Wang Y.-H., Liang C.-W., Li Y.-C. (2019). Assessment of Deep Learning Using Nonimaging Information and Sequential Medical Records to Develop a Prediction Model for Nonmelanoma Skin Cancer. JAMA Dermatol..

[19] Ali L., Zhu C., Zhang Z., Liu Y. (2019). Automated Detection of Parkinson’s Disease Based on Multiple Types of Sustained Phonations Using Linear Discriminant Analysis and Genetically Optimized Neural Network. IEEE J. Transl. Eng. Health Med..

[20] Pereira C.R., Pereira D.R., Rosa G.H., Albuquerque V.H., Weber S.A., Hook C., Papa J.P. (2018). Handwritten dynamics assessment through convolutional neural networks: An application to Parkinson’s disease identification. Artif. Intell. Med..

[21] Caramia C., Torricelli D., Schmid M., Munoz-Gonzalez A., Gonzalez-Vargas J., Grandas F., Pons J.L. (2018). IMU-Based Classification of Parkinson’s Disease From Gait: A Sensitivity Analysis on Sensor Location and Feature Selection. IEEE J. Biomed. Health Inform..

[22] Zhang J. (2022). Mining imaging and clinical data with machine learning approaches for the diagnosis and early detection of Parkinson’s disease. NPJ Park. Dis..

[23] Maass F., Michalke B., Willkommen D., Leha A., Schulte C., Tönges L., Mollenhauer B., Trenkwalder C., Rückamp D., Börger M. (2020). Elemental fingerprint: Reassessment of a cerebrospinal fluid biomarker for Parkinson’s disease. Neurobiol. Dis..

[24] Su C., Tong J., Wang F. (2020). Mining genetic and transcriptomic data using machine learning approaches in Parkinson’s disease. NPJ Park. Dis..

[25] Kang J.-H., Irwin D.J., Chen-Plotkin A.S., Siderowf A., Caspell C., Coffey C.S., Waligórska T., Taylor P., Pan S., Frasier M. (2013). Association of Cerebrospinal Fluid β-Amyloid 1-42, T-tau, P-tau_181_, and α-Synuclein Levels with Clinical Features of Drug-Naive Patients with Early Parkinson Disease. JAMA Neurol..

[26] Silveira-Moriyama L., Carvalho M.d.J., Katzenschlager R., Petrie A., Ranvaud R., Barbosa E.R., Lees A.J. (2008). The use of smell identification tests in the diagnosis of Parkinson’s disease in Brazil. Mov. Disord. Off. J. Mov. Disord. Soc..

[27] Shinde S., Prasad S., Saboo Y., Kaushick R., Saini J., Pal P.K., Ingalhalikar M. (2019). Predictive markers for Parkinson’s disease using deep neural nets on neuromelanin sensitive MRI. NeuroImage Clin..

[28] Armañanzas R., Bielza C., Chaudhuri K.R., Martinez-Martin P., Larrañaga P. (2013). Unveiling relevant non-motor Parkinson’s disease severity symptoms using a machine learning approach. Artif. Intell. Med..

[29] Postuma R.B., Berg D., Stern M., Poewe W., Olanow C.W., Oertel W., Obeso J., Marek K., Litvan I., Lang A.E. (2015). MDS clinical diagnostic criteria for Parkinson’s disease. Mov. Disord. Off. J. Mov. Disord. Soc..

[30] Belvisi D., Pellicciari R., Fabbrini A., Costanzo M., Pietracupa S., De Lucia M., Modugno N., Magrinelli F., Dallocchio C., Ercoli T. (2020). Risk factors of Parkinson disease: Sim-ultaneous assessment, interactions, and etiologic subtypes. Neurology.

[31] Belvisi D., Pellicciari R., Fabbrini G., Tinazzi M., Berardelli A., Defazio G. (2020). Modifiable risk and protective factors in disease development, progression and clinical subtypes of Parkinson’s disease: What do prospective studies suggest?. Neurobiol. Dis..

[32] Wang C., Cai Y., Zheng Z., Tang B.-S., Xu Y., Wang T., Ma J., Chen S.-D., Langston J.W., Tanner C.M. (2012). Penetrance of LRRK2 G2385R and R1628P is modified by common PD-associated genetic variants. Park. Relat. Disord..

[33] Han W., Liu Y., Mi Y., Zhao J., Liu D., Tian Q. (2015). Alpha-synuclein (SNCA) polymorphisms and susceptibility to Parkinson’s disease: A meta-analysis. Am. J. Med. Genet. Part B Neuropsychiatr. Genet. Off. Publ. Int. Soc. Psychiatr. Genet..

[34] Chang X.-L., Mao X.-Y., Li H.-H., Zhang J.-H., Li N.-N., Burgunder J.-M., Peng R., Tan E.-K. (2011). Association of GWAS loci with PD in China. Am. J. Med. Genet. Part B Neuropsychiatr. Genet. Off. Publ. Int. Soc. Psychiatr. Genet..

[35] International Parkinson Disease Genomics Consortium (2011). Imputation of sequence variants for identification of genetic risks for Parkinson’s disease: A meta-analysis of genome-wide association studies. Lancet.

[36] Wang L., Cheng L., Lu Z.-J., Sun X.-Y., Li J.-Y., Peng R. (2016). Association of three candidate genetic variants in RAB7L1/NUCKS1, MCCC1 and STK39 with sporadic Parkinson’s disease in Han Chinese. J. Neural Transm..

[37] Zhao Y., Qin L., Pan H., Liu Z., Jiang L., He Y., Zeng Q., Zhou X., Zhou X., Zhou Y. (2020). The role of genetics in Parkinson’s disease: A large cohort study in Chinese mainland population. Brain J. Neurol..

[38] Prashanth R., Roy S.D., Mandal P.K., Ghosh S. (2016). High-Accuracy Detection of Early Parkinson’s Disease through Multimodal Features and Machine Learning. Int. J. Med. Inform..

[39] Shu Z., Pang P., Wu X., Cui S., Xu Y., Zhang M. (2020). An Integrative Nomogram for Identifying Early-Stage Parkinson’s Disease Using Non-motor Symptoms and White Matter-Based Radiomics Biomarkers from Whole-Brain MRI. Front. Aging Neurosci..

[40] Karabayir I., Butler L., Goldman S.M., Kamaleswaran R., Gunturkun F., Davis R.L., Ross G.W., Petrovitch H., Masaki K., Tanner C.M. (2022). Predicting Parkinson’s Disease and Its Pathology via Simple Clinical Variables. J. Park. Dis..

[41] Lin S., Gao C., Li H., Huang P., Ling Y., Chen Z., Ren K., Chen S. (2023). Wearable sensor-based gait analysis to discriminate early Parkinson’s disease from essential tremor. J. Neurol..

[42] Govindu A., Palwe S. (2023). Early detection of Parkinson’s disease using machine learning. Procedia Comput. Sci..

[43] He S., Huang L., Shao C., Nie T., Xia L., Cui B., Lu F., Zhu L., Chen B., Yang Q. (2021). Several miRNAs derived from serum extracellular vesicles are potential biomarkers for early diagnosis and progression of Parkinson’s disease. Transl. Neurodegener..

[44] Gopar-Cuevas Y., Duarte-Jurado A.P., Diaz-Perez R.N., Saucedo-Cardenas O., Loera-Arias M.J., Montes-De-Oca-Luna R., Rodriguez-Rocha H., Garcia-Garcia A. (2021). Pursuing Multiple Biomarkers for Early Idiopathic Parkinson’s Disease Diagnosis. Mol. Neurobiol..

[45] Mitchell T., Lehéricy S., Chiu S.Y., Strafella A.P., Stoessl A.J., Vaillancourt D.E. (2021). Emerging Neuroimaging Biomarkers across Disease Stage in Parkinson Disease. JAMA Neurol..

[46] Li X., Fan X., Yang H., Liu Y. (2022). Review of Metabolomics-Based Biomarker Research for Parkinson’s Disease. Mol. Neurobiol..

[47] Reichmann H. (2017). Premotor Diagnosis of Parkinson’s Disease. Neurosci. Bull..

[48] Goldman J.G., Postuma R. (2014). Premotor and nonmotor features of Parkinson’s disease. Curr. Opin. Neurol..

[49] Fullard M.E., Morley J.F., Duda J.E. (2017). Olfactory Dysfunction as an Early Biomarker in Parkinson’s Disease. Neurosci. Bull..

[50] Haehner A., Boesveldt S., Berendse H., Mackay-Sim A., Fleischmann J., Silburn P., Johnston A., Mellick G., Herting B., Reichmann H. (2009). Prevalence of smell loss in Parkinson’s disease—A multicenter study. Park. Relat. Disord..

[51] Ponsen M.M., Stoffers D., Booij J., van Eck-Smit B.L.F., Wolters E.C., Berendse H.W. (2004). Idiopathic hyposmia as a preclinical sign of Parkinson’s disease. Ann. Neurol..

[52] Braak H., Ghebremedhin E., Rüb U., Bratzke H., Del Tredici K. (2004). Stages in the development of Parkinson’s disease-related pathology. Cell Tissue Res..

[53] Abbott R.D., Petrovitch H., White L.R., Masaki K.H., Tanner C.M., Curb J.D., Grandinetti A., Blanchette P.L., Popper J.S., Ross G.W. (2001). Frequency of bowel movements and the future risk of Parkinson’s disease. Neurology.

[54] Feng F., Cai Y., Hou Y., Ou R., Jiang Z., Shang H. (2021). Excessive daytime sleepiness in Parkinson’s disease: A systematic review and meta-analysis. Park. Relat. Disord..

[55] Abbott R.D., Ross G.W., White L.R., Tanner C.M., Masaki K.H., Nelson J.S., Curb J.D., Petrovitch H. (2005). Excessive daytime sleepiness and subsequent development of Parkinson disease. Neurology.

[56] Williams-Gray C.H., Mason S.L., Evans J.R., Foltynie T., Brayne C., Robbins T.W., Barker R.A. (2013). The CamPaIGN study of Parkinson’s disease: 10-year outlook in an incident population-based cohort. J. Neurol. Neurosurg. Psychiatry.

[57] Schuurman A.G., Akker M.v.D., Ensinck K.T., Metsemakers J.F., Knottnerus J.A., Leentjens A.F., Buntinx F. (2002). Increased risk of Parkinson’s disease after depression: A retrospective cohort study. Neurology.

[58] Leentjens A.F., Akker M.V.D., Metsemakers J.F., Lousberg R., Verhey F.R. (2003). Higher incidence of depression preceding the onset of Parkinson’s disease: A register study. Mov. Disord. Off. J. Mov. Disord. Soc..

[59] Howell M.J., Schenck C.H. (2015). Rapid Eye Movement Sleep Behavior Disorder and Neurodegenerative Disease. JAMA Neurol..

[60] Postuma R.B., Iranzo A., Hu M., Högl B., Boeve B.F., Manni R., Oertel W.H., Arnulf I., Ferini-Strambi L., Puligheddu M. (2019). Risk and predictors of dementia and parkinsonism in idiopathic REM sleep behaviour disorder: A multicentre study. Brain J. Neurol..

