# Supplementary material for: Applications of Machine Learning to Diagnosis of Parkinson’s Disease
Source: Brain Sci. 2023 Nov 3;13(11):1546. doi: 10.3390/brainsci13111546 (PMC10670005; doi:10.3390/brainsci13111546)
Supplement: Supplementary file 1 [file brainsci-13-01546-s001.zip › brainsci-2660240-supplementary.pdf]

Supplementary Material

Assessment of Potential Risk Factors

Family history of Parkinson's disease (PD) or dementia was assessed using a binary question (yes/no).

Smoking was defined as continuous or cumulative smoking for six months or more. Alcohol consumption was characterized as drinking at least once per month. Coffee and tea consumption were defined as consuming one cup or more per week.

History of exposure to pesticides, including insecticides and herbicides, was evaluated using a yes/no question. Occupational exposure to toxicants, such as organic solvents and heavy metals, was defined as work exposure to these materials for more than six months and assessed through a binary question. Histories of head injury were defined as head injuries resulting in a loss of consciousness. For anesthesia, subjects were asked to recall whether they had undergone any general anesthesia (yes/no).

Standardized scales were utilized to assess motor and non-motor symptoms. The Movement Disorders Society Unified Parkinson Disease Rating Scale part III (MDS-UPDRS-III) was employed for standardized motor examination [1]. Possible REM sleep behavior disorders were assessed using the RBD Screening Questionnaire (RBDSQ), with a cut-off value of 6 on the RBDSQ score [2]. Olfactory dysfunction was evaluated using the Hyposmia Rating Scale (HRS), with a cut-off value of 23 on

the HRS score [3]. Constipation was assessed using the Rome III criteria, with a diagnosis of functional constipation in accordance with two or more items [4]. Global cognitive deficits were evaluated using the Mini-Mental State Examination (MMSE), with a cut-off value of 27 on the MMSE score [5]. Depression was assessed using the 17-item Hamilton Depression Scale (HAMD), with a cut-off value of 8 on the HAMD score [6]. Daytime somnolence was evaluated using the Epworth Sleepiness Scale (ESS), with a cut-off value of 10 on the ESS score [7].

Machine Learning Algorithm

The Least Absolute Shrinkage and Selection Operator–Logistic Regression (LASSO-LR) method constructs a logistic regression model by retaining variables through more stringent thresholds, thereby selecting a parsimonious, predictive subset of variables to train the model. To address multiple cross-related covariates and reduce the risk of overfitting, LASSO-LR with 10-fold cross-validation was employed for identifying the most useful predictive factors from the training set [8]. An L1 norm was added as a penalty in calculating the minimum residual sum of squares (RSS). As the lambda increases, some coefficients can be accurately reduced to zero. We then selected the lambda of 1 standard, where the cross-validation error is minimized [8,9].

Parameter Interpretation

Lambda (λ): This is the regularization parameter in LASSO regression. It controls the

strength of the penalty imposed on the coefficients in the model. A larger λ value will cause more coefficients to tend towards zero, producing a sparser model. Selecting the appropriate λ value is vital, as it directly impacts the model's complexity and predictive capability.

Decision Tree

A decision tree is an inverted tree structure consisting of root nodes, inner nodes, leaf nodes, and edges. The root node is the uppermost node, the leaf node provides the judgment conclusion, and the internal node is a relative concept. The purpose of constructing a decision tree is classification or regression.

Parameter Interpretation

Complexity Parameter (CP): This is a parameter utilized for pruning. When a node's split does not result in a significant improvement in the model's fit, that node will not be split. A larger CP value produces a smaller tree, simplifying the model.

Random Forest

A random forest (RF) is a combination of decision trees [10]. Each tree is generated through bootstrapping sampling, and splits are performed at each node using a randomly sampled predictor set. Due to the random subsets of features selected at each node, RF is robust to high correlations between inputs. Tuning parameters include the number of predictor variables selected by each node (mtry) and the

number of sampling trees (ntrees).

Parameter Interpretation

Number of Trees: This determines the number of decision trees within the random forest. Increasing the number of trees can enhance the model's stability and accuracy but also augments the computational complexity.

mtry: This represents the number of features randomly chosen at each split. A smaller mtry value can boost the model's diversity but might also increase the model's variance.

EXtreme Gradient Boosting

The eXtreme gradient boosting (XGboost) algorithm, introduced in 2014, builds upon the boosting algorithm and has demonstrated exceptional performance and usability across numerous data science competitions. By offering solutions with the potential to significantly enhance decision-making efficiency and quality, as well as reduce false positive rates, XGboost can automatically discern the optimal splitting direction when managing missing values. Furthermore, the algorithm can efficiently and accurately emulate nonlinear effects when processing large datasets [11].

Parameter Interpretation

Learning Rate (eta): This controls the adjustment step size in each iteration. A smaller learning rate can make the model more robust but might necessitate more iterations.

colsample_bytree: This determines the fraction of features used when constructing each tree, aiding in preventing overfitting.

Gamma: This controls the depth and size of the trees. A larger gamma value results in smaller trees.

Max_depth: This restricts the maximum depth of the tree, assisting in preventing overfitting.

Subsample: This determines the fraction of data used for training each tree, also helping in preventing overfitting.

Min_child_weight: This controls the minimum sum of instance weight (hessian) needed in a child (leaf).

Support Vector Machine

The support vector machine (SVM) is particularly well-suited for small datasets. It classifies data into groups by identifying hyperplanes that optimally separate the dataset [12]. One crucial hyperparameter is the kernel, which can be linear or nonlinear (e.g., radial, sigmoid, or polynomial) and projects data into multidimensional space to enhance classification. Another essential aspect is the cost function, which represents the penalty assigned to data within the margin of error. Higher values result in lower bias but increased variance.

Parameter Interpretation

Cost (C): This is a regularization parameter determining the penalty for misclassified samples. A larger C value can make the model more complex, potentially leading to overfitting.

Kernel Smoothing Parameter (σ): This parameter is used for non-linear SVMs and determines the distribution of data in the feature space.

K-Nearest Neighbor

The k-nearest neighbor (KNN) algorithm classifies new datapoints by leveraging information from the datapoints closest to it [13]. The most likely classification is determined by the majority vote among the KNN. The k parameter is adjusted to identify the optimal number of neighbors for maximum predictive power.

Parameter Interpretation

Number of Neighbors (k): This determines the number of neighbors used for classification. A smaller k value can make the model more sensitive to noise, while a larger k value might oversimplify the model.

References

[1] C.G. Goetz, B.C. Tilley, S.R. Shaftman, G.T. Stebbins, S. Fahn, P. Martinez-Martin, et al., Movement Disorder Society-sponsored revision of the Unified Parkinson's Disease Rating Scale (MDS-UPDRS): scale presentation and clinimetric testing results. Mov Disord 23 (2008) 2129-70. http://doi.org/10.1002/mds.22340

[2] P. Millar Vernetti, S. Perez Lloret, M. Rossi, D. Cerquetti, and M. Merello, Validation of a new scale to assess olfactory dysfunction in patients with Parkinson's disease. Parkinsonism Relat Disord 18 (2012) 358-61. http://doi.org/10.1016/j.parkreldis.2011.12.001

[3] T. Nomura, Y. Inoue, T. Kagimura, Y. Uemura, and K. Nakashima, Utility of the REM sleep behavior disorder screening questionnaire (RBDSQ) in Parkinson's disease patients. Sleep Med 12 (2011) 711-3. http://doi.org/10.1016/j.sleep.2011.01.015

[4] D.A. Drossman, Rome III: The Functional Gastrointestinal Disorders, 2006. http://doi.org/doi:10.3748/wjg.14.2124

[5] M.F. Folstein, S.E. Folstein, and P.R. McHugh, "Mini-mental state". A practical method for grading the cognitive state of patients for the clinician. J Psychiatr Res 12 (1975) 189-98. http://doi.org/10.1016/0022-3956(75)90026-6

[6] M. Zimmerman, J.H. Martinez, D. Young, I. Chelminski, and K. Dalrymple, Severity classification on the Hamilton Depression Rating Scale. J Affect Disord 150 (2013) 384-8. http://doi.org/10.1016/j.jad.2013.04.028

[7] M.W. Johns, A new method for measuring daytime sleepiness: the Epworth sleepiness scale. Sleep 14 (1991) 540-5. http://doi.org/10.1093/sleep/14.6.540

[8] J. Friedman, T. Hastie, and R. Tibshirani, Regularization Paths for Generalized Linear Models via Coordinate Descent. J Stat Softw 33 (2010) 1-22.

[9] R. Tibshirani, The lasso method for variable selection in the Cox model. Stat Med 16 (1997) 385-95. http://doi.org/10.1002/(sici)1097-0258(19970228)16:4<385::aid-sim380>3.0.co;2-3

[10] L. Breiman, Random Forests. Machine Learning 45 (2001) 5-32. http://doi.org/10.1023/A:1010933404324

[11] T. Chen, and C. Guestrin, Xgboost: A scalable tree boosting system, Proceedings of the 22nd acm sigkdd international conference on knowledge discovery and data mining, 2016, pp. 785-794. https://doi.org/10.1145/2939672.2939785

[12] C. Cortes, and V. Vapnik, Support-vector networks. Machine Learning 20 (1995) 273-297. http://doi.org/10.1007/BF00994018

[13] J.M. Keller, M.R. Gray, and J.A. Givens, A fuzzy K-nearest neighbor algorithm. IEEE Transactions on Systems, Man, and Cybernetics SMC-15 (1985) 580-585. http://doi.org/10.1109/TSMC.1985.6313426

Table S1 The primer sequences for amplifying each SNPs

Gene	SNPs	1st-sequence of PCR primer	2st-sequence of PCR primer
MMRN1	rs6532194	ACGTTGGATGCTTCCCTATTTGTAGAGTGC	ACGTTGGATGGGCATTGAACAAAGCATGGG
RAB7L1	rs823144	ACGTTGGATGAATGTAGTGCCACAGGCAAC	ACGTTGGATGCCAAATTGTGAGGAAGGAGC
SNCA	rs356182	ACGTTGGATGCTCTTCCTCTCCCTGGAGAA	ACGTTGGATGGCATCTGGTGCATCTATTCC
	rs356129	ACGTTGGATGCATGGGTATACTGGTGGTTC	ACGTTGGATGGCTGTGGGAAACAAGGTACT
LRRK2	rs34778348	ACGTTGGATGGCAAAATAGCCCTGTTGTGG	ACGTTGGATGCTTAAAAAGTGCACGCAGTC
MCCC1	rs12637471	ACGTTGGATGAACCACATCACCTCAAGGTC	ACGTTGGATGTGAAACCGAATGATGGCCTG
GBA	rs421016	ACGCTGTCTTCAGCCCACTTC	GGAGGACCCAATTGGGTGCGT

Figure legends

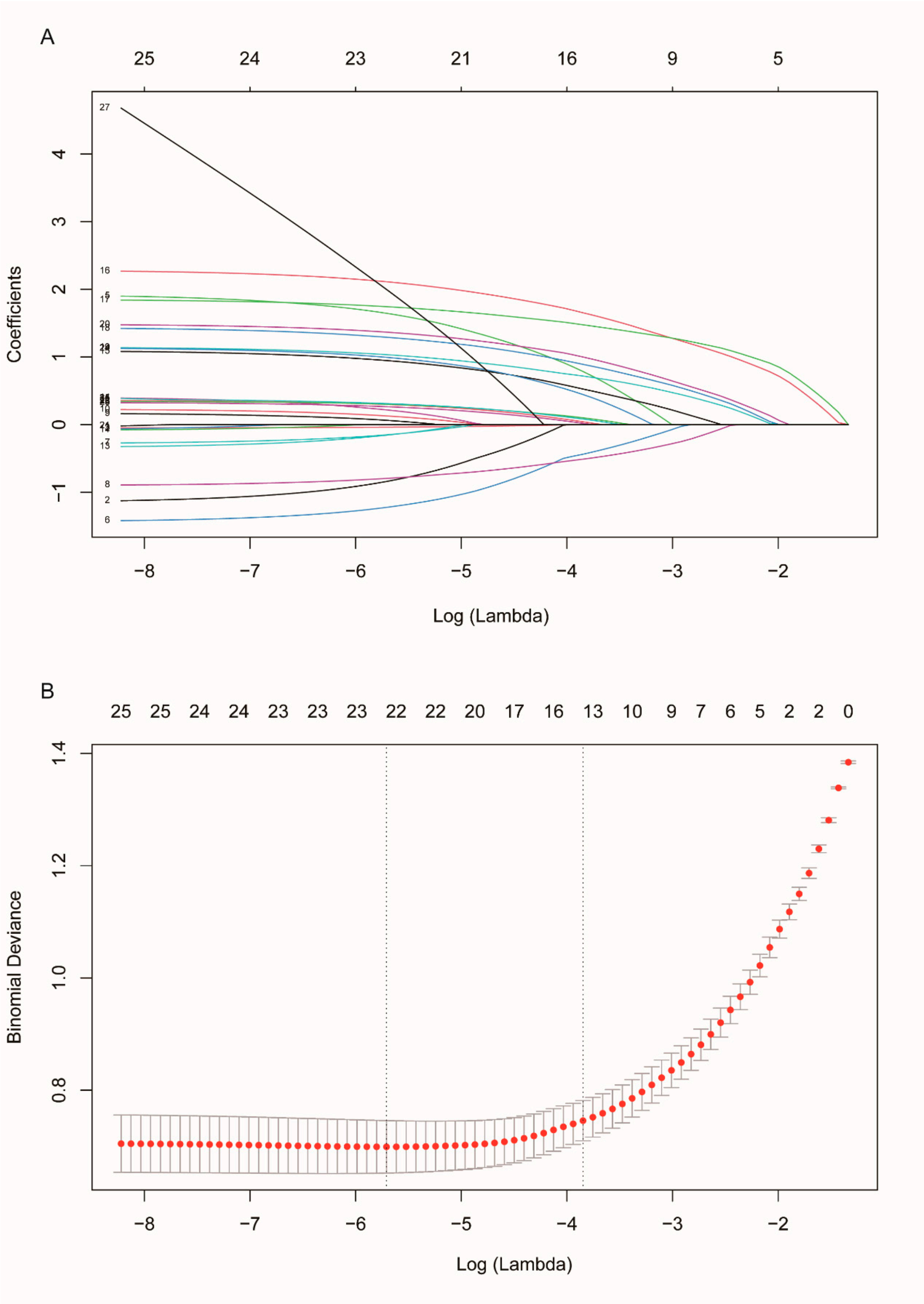

Figure S1 Feature selection using Lasso regression. (**A**) Lasso coefficient profiles of the candidate features. (**B**) Selection of the optimal parameter (lambda) through 10-fold cross-validation. The left and right dotted vertical lines represent the optimal

lambda values using the minimum error criterion and one standard error (1-SE) of the minimum criterion, respectively.

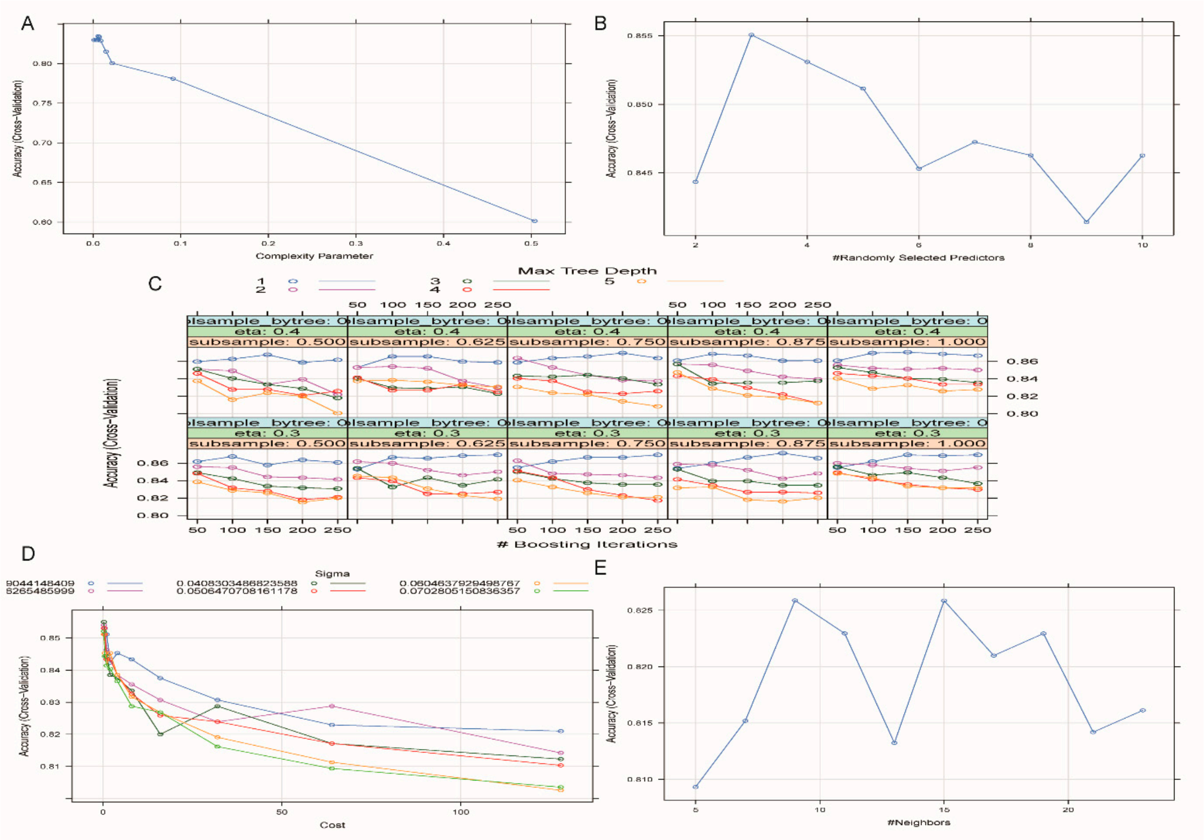

Figure S2 Hyperparameter optimization for each machine learning model using five-fold cross-validation and a grid search technique. (**A**) Hyperparameter optimization for the decision tree model. (**B**) Hyperparameter optimization for the random forest model. (**C**) Hyperparameter optimization for the eXtreme gradient boosting model. (**D**) Hyperparameter optimization for the support vector machine model. (**E**) Hyperparameter optimization for the k-nearest neighbor model.
